# Recycling of Clay Sediments for Geopolymer Binder Production. A New Perspective for Reservoir Management in the Framework of Italian Legislation: The Occhito Reservoir Case Study

**DOI:** 10.3390/ma7085603

**Published:** 2014-07-31

**Authors:** Bruno Molino, Annamaria De Vincenzo, Claudio Ferone, Francesco Messina, Francesco Colangelo, Raffaele Cioffi

**Affiliations:** 1 Department of Biotechnology and Territory, University of Molise, Campobasso 86100, Italy; E-Mail: bruno.molino@unimol.it; 2 School of Engineering, University of Basilicata, Potenza 85100, Italy; E-Mail: annamaria.devincenzo@unibas.it; 3 Department of Engineering, University of Naples “Parthenope”, Centro Direzionale Isola C4, Naples 80143, Italy; E-Mails: claudio.ferone@uniparthenope.it (C.F.); colangelo@uniparthenope.it (F.C.); raffaele.cioffi@uniparthenope.it (R.C.)

**Keywords:** sustainability, reservoir, silting-up, sediments, geopolymers, binders

## Abstract

Reservoir silting is an unavoidable issue. It is estimated that in Italy, the potential rate of silting-up in large reservoirs ranges from 0.1% to 1% in the presence of wooded river basins and intensive agricultural land use, respectively. In medium and small-sized reservoirs, these values vary between 0.3% and 2%. Considering both the types of reservoirs, the annual average loss of storage capacity would be of about 1.59%. In this paper, a management strategy aimed at sediment productive reuse is presented. Particularly, the main engineering outcomes of an extensive experimental program on geopolymer binder synthesis is reported. The case study deals with Occhito reservoir, located in Southern Italy. Clay sediments coming from this silted-up artificial lake were characterized, calcined and activated, by means of a wide set of alkaline activating solutions. The results showed the feasibility of this recovery process, optimizing a few chemical parameters. The possible reuse in building material production (binders, precast concrete, bricks, *etc.*) represents a relevant sustainable alternative to landfill and other more consolidated practices.

## 1. Introduction

### 1.1. Italian Legislation Framework

In Italy, there are about 555 large dams, according to the definition of [1], where the “large dams” are the ones higher than 15 m with a total storage capacity greater than 1 × 10^6^ m^3^. Among them, 494 are still in operation, together with a number of small dams that, according to different estimations, range from 8288 to 15,400. Large and small dams have an overall potential storage capacity of approximately 10,854 × 10^6^ m^3^ and 300 × 10^6^ m^3^, respectively.

The overall capacity of the reservoir is a poorly renewable resource, and the environmental problems resulting from the construction of new dams significantly reduces the number of areas suitable for the realization of new reservoirs. This is true for both large and small-sized reservoirs.

It is estimated that in Italy, the potential rate of silting-up in large reservoirs ranges from 0.1% to 1% in the presence of wooded river basins and intensive agricultural land use, respectively. In the reservoirs of medium and small size, these values vary between 0.3% and 2%. Considering both the types of reservoirs, the annual average loss of storage capacity would be about 1.59% [2].

An analysis performed on 13 large reservoirs located in Sicily showed a significant loss of storage capacity due to the deposit processes, with a consequent decreasing of the overall storage capacity from 489 to 432 × 10^6^ m^3^ and an overall loss of about 12% of the initial storage volume [3,4].

The analysis carried out by [5] on a sample of 268 large dams built in Italy in the last 50 years has evidenced that already 20 years ago:

- 1.5% of the dams were almost completely filled by sediments;
- 17.5% showed a reservoir capacity reduction of more than 50%;
- 4.5% showed a reservoir capacity reduction of approximately 20%.

In addition to the high costs of desiltation measures, it is essential to point out that sediments removed from a reservoir bottom are essentially under anaerobic conditions, with high concentrations of soluble iron and manganese (reduced forms), S^2−^, S^−^ and H_2_S [6,7]. Therefore, they are highly toxic to plants and can cause strong environmental impacts [5]; therefore, as a temporary remedy, they are often moved from the area close to the dam, where they can interfere with the hydraulic works, and deposited in a new area within the same reservoir.

The management of reservoir siltation aimed at the maintenance of useful storage capacity is a matter of Legislative Decree 152/2006 (Article 114) and subsequent amendments [8]. Article 114 requires the preparation of a Management Plan for each reservoir, prepared on the basis of the criteria established by decree of the Ministry of Infrastructures and Transport, and the Environmental and Territorial Protection with the advice of the Ministry of Productive Activities and the Ministry of Agriculture and Forestry. At present, the decree has not been issued yet and, according to [9], it will be needed therefore to refer to the Ministerial Decree of 30 June 2004 [10].

Currently, also, Article 43 of the Decree Law, 201/2011, and subsequent amendments provides for the obligation to draw up the Management Project of large dams on the basis of which the competent authorities (Ministry of Infrastructures and Transport, Autonomous Regions and Provinces) will identify the plants for which the removal of bottom sediment is necessary [11].

Article 1, Paragraph 2 of the [10], provides that the Italian Regions establish specific regulations for reservoirs, which are not subject to the provisions of the decree of the President of the Republic 1363/59 and for the pertinent water bodies (Small Reservoirs) [12].

At present, some Italian Regions have already enacted regional regulations for dams not subject to the requirements of [12], as provided by the Article 1, Paragraph 2 of [10]. These regulations are the only source of information and technical guidance for several specific issues.

In the current Italian legislative framework, sediments, once removed from the reservoir bottom, can be:

- disposed of in a landfill as waste according to [13];
- recovered under the normal or simplified procedure specific for dredging according to [14];
- reused as a by-product, according to Article 184 of [8] and subsequent amendments (Legislative Decree 205/2010) [15].

Excavation soils should be regarded as being a by-product and not waste only when certain conditions are met, such as:

(a) excavation soils result from the completing of a work and are part of a production process whose primary purpose is not the production of such material;
(b) excavation soils are used in accordance to the Use Plan;
(c) the excavation soils can be directly used without any further processing other than normal industrial practice (Annex 3 to the Ministerial Decree, 161/2012) [16];
(d) for the specific use to which Point (c) is refers, the excavation soils fulfil the environmental protection requirements specified in Annex 4 to [16].

One of the critical issues in this decree concerns the lack of a simplified procedure for small yards where soil production does not exceed 6000 m^3^ (as specifically provided for by Article 266, Paragraph 7 of [8]).

In this sense, some Italian Regions have issued *ad hoc* regional laws, for instance:

- Liguria Region, Regional Council Decree No. 3159 of 01 February 2013 [17];
- Veneto Region, Regional Council Decree No. 179 of 11 February 2013 [18].

With reference to the sediments of a reservoir, based on the present state of scientific knowledge, the problem is to extend the life cycle of the dredged sediments by their sustainable reuse solutions [19].

In [20], the idea of the “economic defense” of a reservoir was presented, which combines reservoir rehabilitation and reservoir utilization for environmental, industrial and agricultural purposes. In this sense, the economic defense, which was introduced as a form of evolution of the reservoir sedimentation passive defense, involves also an economic benefit. In the next sections, this innovative perspective will be applied to the case study of Italian Occhito Reservoir, by considering geopolymers binder production as a management strategy oriented toward the “economic defense” paradigm.

### 1.2. Geopolymer Binders as an Innovative Management Strategy

The evolution of national and European legislations in the field of reservoir water and sediment management was a strong push factor for scientific research towards the determination of possible reuses of sediment [21,22].

Some interesting results were obtained in the following works concerning reservoir sediments:

- use of harbor, river and reservoir sediments in brick production [23,24,25];
- use of harbor and reservoir sediments in sintered lightweight aggregate manufacturing [26,27,28,29,30,31];
- use of clay sediment for the production of Portland cement [32];
- use of silt-clay sediment for agriculture amendment [33];
- use of sandy sediments for beach nourishment and protection from erosion [34].

Concerning the beneficial reuse of sediments, two main research trends can be found: bricks [23,24,25] and sintered lightweight aggregates [26,27,28,29,30,31]. In both cases, high temperature industrial processes are required, implying a detrimental environmental effect. Lightweight aggregates could be obtained with alternative low temperature processes, such as cold bonding pelletization [35].

In this work, an extensive experimental program is presented in order to find the best management solution to employ reservoir sediments in the production of geopolymers. A remarkable growth of research in the field of geopolymers has been observed in the last fifteen years. This growth has been pushed first by the increasing pulse toward sustainability, which has involved also the construction sector. Furthermore, there have been highly promising results obtained by some pioneering research in terms of durability, fire resistance, *etc.* [36,37,38,39]. All of these factors have determined also new industrial applications, which have given a second development of geopolymers technology after those considered as forerunners in Belgium, Eastern Europe, China, *etc.* [40]. Even if many potential applications have been investigated in the literature [41,42], use in civil engineering works still remains the main market [43,44].

In a preliminary study [45], sediments coming from two reservoirs located in Southern Italy were characterized by means of several chemical tests, revealing the potential to be employed as raw materials for the synthesis of geopolymers after calcination at 650 °C and 750 °C. The effectiveness of this activation treatment increased with temperature. However, just one mix design was considered (5 M NaOH solution).

In this study, the abovementioned preliminary results were qualitatively and quantitatively compared with a broader set of binding mixtures, considering potassium and sodium aluminate solutions, with different concentrations. Furthermore, in this case, the experimental program relied on a more significant sample made by homogenizing ten cores randomly taken from the lake. 

In the following sections, the optimization of binding systems synthesized with Occhito sediments and characterization procedures will be accurately described, and the main engineering outcomes and impacts on reservoir management will be discussed.

## 2. Results and Discussion

XRF analysis results are shown in Table 1. The results of the leaching test are not shown here, but they confirmed that the Occhito sediment can be classified as non-hazardous wastes. The code associated with this residue in the European Waste Catalogue is 17.05.06 (sludge dredging).

**Table 1:** XRF analysis of Occhito sediment.

Oxides	SiO_2_	Al_2_O_3_	CaO	Fe_2_O_3_	MgO	K_2_O	TiO_2_	Na_2_O	MnO	P_2_O_5_
Occhito	57.13	16.33	12.5	6.94	2.97	2.4	0.88	0.37	0.15	0.15
MK1	51.2	43.98	-	1.12	-	0.54	1.67	-	-	-
MK2	52.9	41.9	0.17	1.6	0.19	0.77	1.8	-	-	-

In Table 1 are also reported two commercial metakaolin samples, namely MK1 and MK2. As we can immediately observe, the clay coming from reservoir dredging is not a pure aluminosilicate solid precursor. Particularly, the alumina content is sensitively lower than in the cases of commercial metakaolin samples. Nevertheless, the high CaO content, if amorphous, can influence polycondensation, modifying the mineralogy of hardened samples by determining the presence of calcium silicate hydrate-based phases [46]. Iron, magnesium and potassium contents can also determine complex mineralogical systems in the matrix. However, the Occhito sample as received is unreactive and, so, unsuitable for geopolymer binder production. In the next lines, the effect of the calcination processes carried out at 650 and 750 °C will be discussed.

The results of semiquantitative analysis on Occhito sediments revealed that clay phases represent the main crystalline component (65%). Significantly lower contents of quartz (18%) and calcite (17%) were also detected, while a limited amount of feldspar (3%) was found [45].

**Figure 1:**
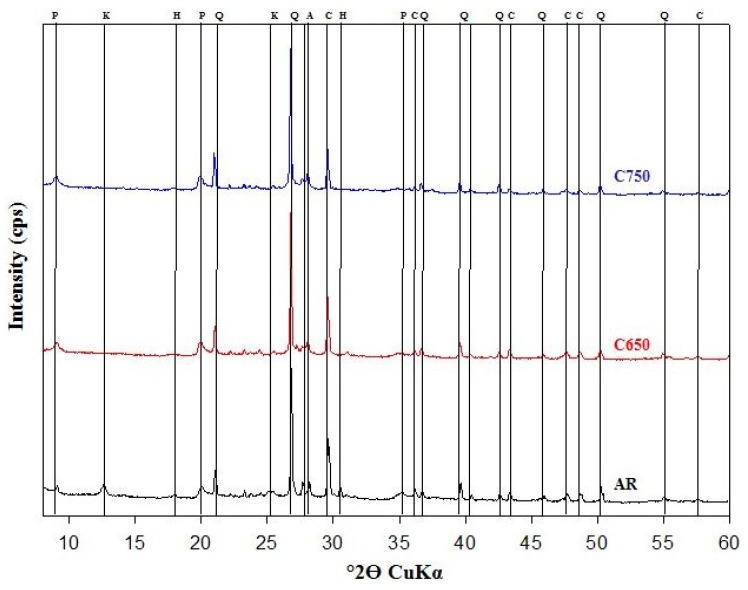
Mineralogical composition of Occhito sediments as received (indicated with “AR”), calcined at 650 °C (C650) and 750 °C (C750).

In Figure 1, XRD results are shown. The main detected mineralogical phases were: anorthite (A, CaO·Al_2_O_3_·2SiO_2_); calcite (C, CaCO_3_); hillebrandite (H, 2CaO·SiO_2_·H_2_O); kaolinite (K, Al_2_O_3_·2SiO_2_·2H_2_O); paragonite, (P, NaAl_2_ [Si_3_Al]O_10_(OH)_2_); quartz (Q, SiO_2_). The Occhito sample as received showed a mineralogical composition, which is mainly characterized by clay minerals, quartz and calcite. Iron, magnesium and potassium contents found by means of XRF did not find correspondent mineralogical phases in XRD, revealing that these elements could be present in amorphous phases. Thermal treatment determines the dehydration of clay minerals with the formation of reactive components.

**Figure 2:**
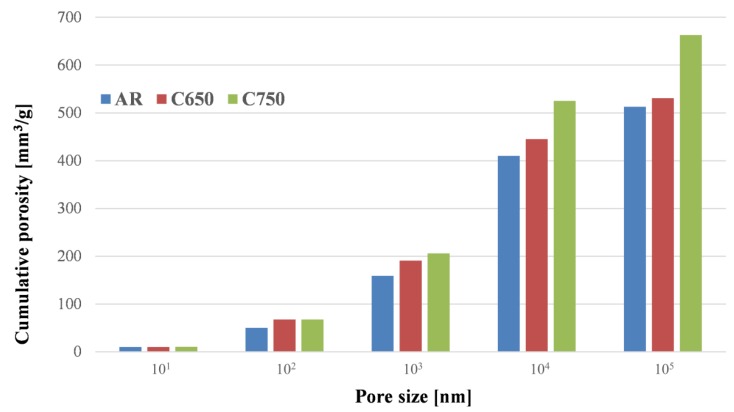
Mercury intrusion porosimetry results.

The physical effect of calcination was investigated by means of MIP (Figure 2). Five pore ranges were observed (10^1^–10^5^ nm), revealing an increase of porosity in calcined samples starting from 10^3^ nm-sized pores. The total porosity values were 513, 531 and 664 mm^3^/g for as-received and calcined at 650 °C and 750 °C samples, respectively.

**Figure 3:**
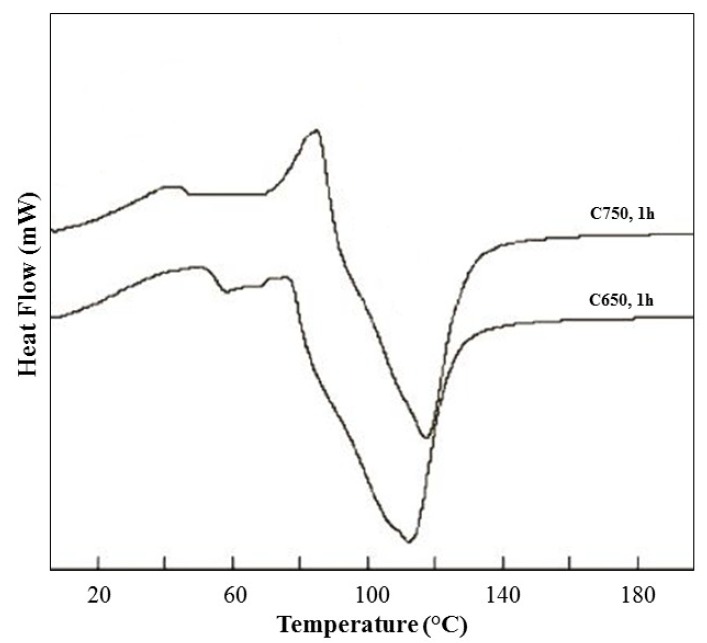
DSC analysis results.

In Figure 3, the results of DSC analysis are shown. As outlined in previous sections, a dynamics test was carried out. The main outcomes of this experimental step were: (1) the exothermic peaks area was higher when calcination temperature increases; (2) the highest peaks were detected around 60 °C. The latter consideration is the reason for setting the curing temperature at 60 °C.

**Figure 4:**
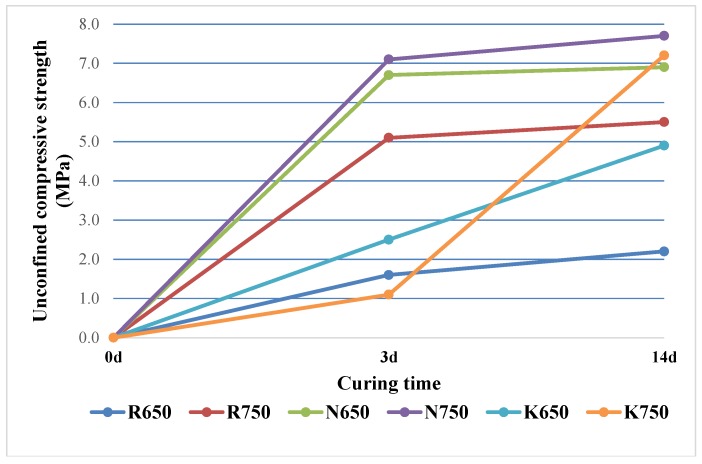
Unconfined compressive strength values after three and seven days of curing.

In Figure 4, the results of mechanical testing are reported. Strength development was monitored for 14 days. The curves indicated by R650 and R750 represent the reference systems, obtained by activating calcined samples by means of 5 M NaOH solution. These systems exhibited a strong dependence on calcination temperature. Passing from 650 °C to 750 °C, we observed an increase in the early strength by a factor of three. In both cases of R650 and R750, the curing phase carried out under room conditions did not determine a further strength increase.

The qualitative analysis revealed that a proper mechanical performance development was achievable only in the cases of the 13 M sodium aluminate solution and 11 M potassium aluminate solution. Compressive strength development is reported here for brevity only for four aluminate-activated systems (namely N650, N750, K650, K750).

In Figure 4, the different influence of sodium and potassium aluminate solutions on hardened products strength is also observable. Samples obtained by activating calcined Occhito sediments with potassium aluminate, namely K650 and K750, exhibited a peculiar behavior. In the early age, calcination temperature seemed to bring no benefit to strength development, since the K650 compressive strength was higher than K750 by a factor of about 2.5. The situation is completely reversed after 14 days, when K750 samples show a tripled strength compared to K650 ones. Anyway, the geopolymers made with sediment calcined at 750 °C (K750) were able to develop relevantly the mechanical properties also at room conditions, while K650 showed a lower increase after the first three days at 60 °C. The systems activated with sodium aluminate showed the best mechanical performances. Furthermore, the kinetics were only slightly improved by increasing calcination temperature, as we can see from Figure 4. This is of course an interesting result, in terms of both performance development and sustainability indicators.

**Figure 5:**
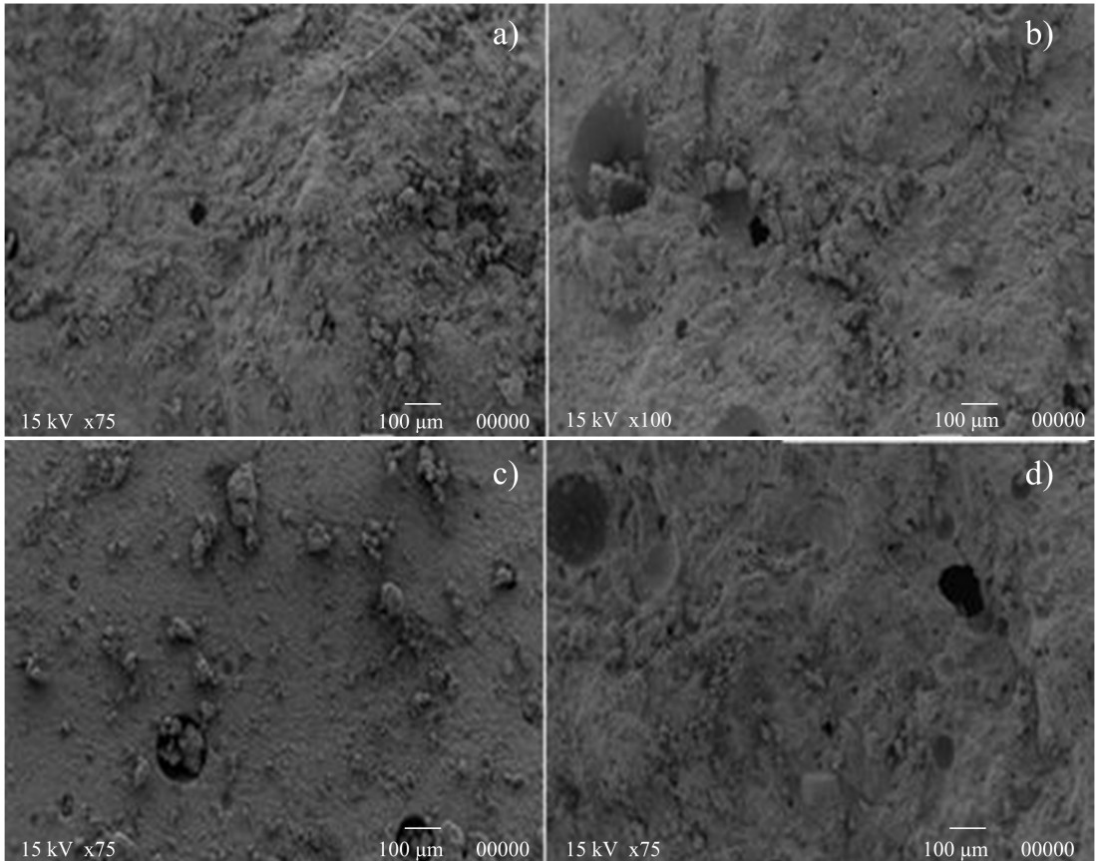
Microstructural analysis of N650 (**a**,**c**) and N750 (**b**,**d**) samples.

In Figure 5a,c, SEM images of N650 samples after three and 14 curing days are reported. As we can observe, no substantial microstructural changes occurred during the longer curing phase at room temperature. This result is in agreement with strength development, since no significant increase of compressive resistance was observed. A similar conclusion can be drawn considering Figure 5b,d, which is related to N750 samples after three and 14 curing days.

**Figure 6:**
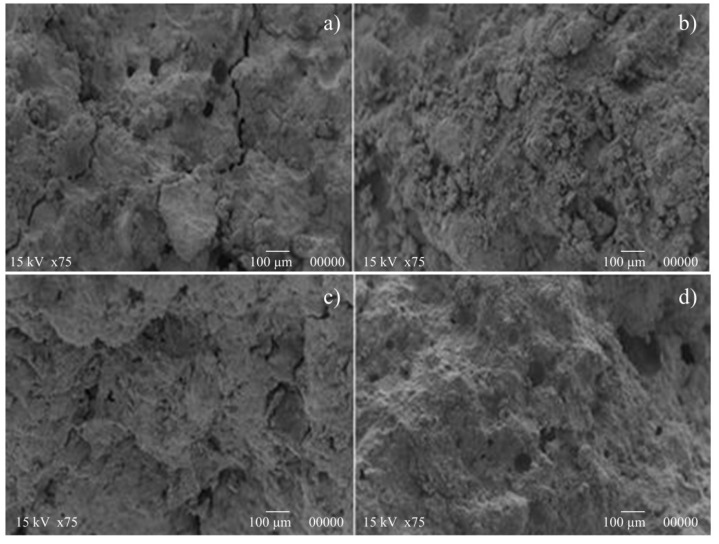
Microstructural analysis of K650 (**a**,**c**) and K750 (**b**,**d**) samples.

Figure 6 reports SEM images for K650 and K750 samples. The microstructure at the early age (after three days of curing) can be observed in Figure 6a (K650) and Figure 6b (K750). These observations explain the unsatisfactory mechanical performance obtained, since relevant microcracking patterns and poorly compacted microstructures were developed at the early age. Curing time definitely influences microstructure refinement only in the case of K750, with the development of a more compact microstructure (Figure 6d). Instead, in the case of K650, the presence of microcracks limits the strength gain, determining the premature brittle failure of the sample under testing. These results are in agreement with the outcomes of mechanical testing.

## 3. Experimental Section

The samples of clay sediments were collected from the Italian reservoir of Occhito (Carlantino, Foggia, Italy). Many samples were obtained by coring, obtaining ten cores from Occhito Lake. The cores were 10 cm in diameter and 1 m in height. The cores were first crushed in a jaw crusher; then, they were milled in a ring mill and carefully mixed to get a homogeneous sample.

The sample was dried in an oven at 105 °C and then submitted to quantitative chemical analysis. The composition in terms of main oxides was obtained by means of X-ray fluorescence, making use of a Bruker Explorer S4 apparatus. Calcination was carried out at 650 °C and 750 °C for 1 h. The mineralogical analysis was performed by means of a Philips PW 1730 diffractometer (Cu *K_α_* radiation, 5°–60° 2*ϴ* range, step width of 0·02° 2*ϴ* and 1 s data collection per step).

Semiquantitative evaluation of the mineralogical phases was performed according to the following conditions: powders with a grain size <10 µm have been obtained by means of a McCrone micronizing mill (agate cylinders and wet grinding time = 15 min). An α-Al_2_O_3_ internal standard (1 µm, Buehler Micropolish) has been added to each sample in the amount of 20 wt%. Powder data sets were obtained with a PANalytical X’Pert Pro modular diffractometer equipped with an RTMS detector (Cu *K_α_* radiation, 40 kV, 40 mA, 2θ range from 3° to 80°, equivalent step size of 0·0179° 2θ, equivalent counting time of 120 s per step). X’Pert High Score Plus 2·2d software and the inorganic crystal structure database (ICSD) were used for mineral identification and semiquantitative evaluation.

In order to assess the physical effect of calcination, mercury intrusion porosimetry (MIP) testing was carried out on both as-received and calcined samples. The pore structure of powder is strictly related to the specific surface, and any variation can influence reactivity. The experimental equipment consisted of two porosimeters apparatus, namely Thermo Pascal 140 and Thermo Pascal 440, which reach a maximum pressure of 400 kPa and 400 MPa, respectively. The pore shapes are assumed to be cylindrical, and their diameters can be calculated by the Washburn equation:

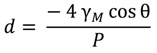
(1)
In this formula, *d* is the pore diameter (m); γ_M_ is the mercury surface tension (N/m); *P* is the applied pressure (MPa); and ʘ is the contact angle between the mercury and the solid surface (°).

Calcined clay samples dredged from Occhito Lake were employed in geopolymer binder preparation. The case study presented here was concerned with the optimization of the binding mixture by means of several alkaline activating solutions. Particularly, considering the low content of alumina in the raw sediments, it was suggested to design binding systems with alumina-containing activating solutions. Most of the literature study cases consider aluminosilicate precursors activated by means of alkaline hydroxide/silicate solutions for geopolymers synthesis. In the case of this experimental work, where sediments showed a low alumina content, the use of alkaline aluminate solutions appeared to be a very interesting solution. In the literature, only a small amount of data about aluminate-based activation of geopolymers can be found. Nevertheless, the results obtained with the aluminate option were considerably promising. In [47], a separate mixing procedure was applied to a silica fume-based geopolymer system activated by means of: (1) sodium hydroxide solution; (2) sodium aluminate solution. The hardened binder samples exhibited good agreement with the geopolymer science criteria, e.g., they were X-ray amorphous, set and hardened in mild conditions, achieving fine mechanical performance (about 26 MPa). The only crystalline phase detected was gibbsite, mainly attributable to the carbonation of 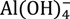 species [47]. Hajimohammadi *et al.* [48] studied a one-part geopolymer composition obtained by adding water to a solid geothermal silica and sodium aluminate blend, but engineering characterization was not carried out. In [49], many binding mixtures were studied and, among those, sodium aluminate activated kaolinite, slag, K-feldspar and fly ash mixtures. The alumina content obtained by means of XRF analysis was equal to 28.6, 17.6, 14.1 and 27.1 wt% for kaolinite, k-feldspar, slag and fly ash, respectively. Compressive strength values after 7 days of curing ranged from 3.3 to 26.6 MPa when only sodium aluminate was used and from 23.0 to 86.5 MPa when a binary aluminosilicate alkaline solution was used [49]. Recently, other works have demonstrated the interest of research in aluminate activation. Rickard *et al.* investigated the thermal properties of fly ash-based geopolymers activated by means of sodium silicate and sodium aluminate solutions [50]. Van Riessen *et al.* obtained average performance geopolymers (compressive strength in the range 33–43 MPa) activating fly ash by means of industrial liquors coming from the Bayer process [51]. All of these considerations reasonably push research toward the further development of aluminate activation, which could bring not just average performance composites for the construction sector, but also high performance ones.

In this work, three different series of alkaline solutions were employed:

(1) 5 M NaOH in an amount so as to have a Na/Al ratio at most equal to 1:1; this is possible by assuming the hypothesis that all of the alumina is available for polycondensation reactions;
(2) sodium aluminate solution obtained by dissolving pure Al(OH)_3_ in NaOH solutions in an amount so as to have a molar Al/Na ratio equal to 0.65 (maximum solubility);
(3) potassium aluminate solution obtained by dissolving pure Al(OH)_3_ in KOH solutions in an amount so as to have a molar Al/K ratio equal to 0.53 (maximum solubility).

In both of the two latter cases, several alkaline solution concentrations were evaluated by means of a qualitative inspection of hardened samples. Particularly, sodium aluminate solutions with concentration of 8.5, 11, 13, 15 and 17 and potassium aluminate solutions with a concentration of 8.5, 11, 13 and 17 were considered.

The reactivity of samples was tested by measuring polycondensation occurrence through a differential scanning calorimetry (DSC) apparatus, TA Instruments model Q20, according to the following experimental details: the reactants (calcined sediments and 5 M NaOH solution) were separately kept at 4 °C and intimately mixed for 3 min before testing. Particularly, a dynamics test was carried out on a sample of 10 mg by heating up to 200 °C at 5 °C min^−1^. The reactivity was evaluated by measuring the heat evolved in each experimental condition, assessing in this way the effect of calcination temperature on polycondensation.

In order to verify the actual occurrence of geopolymerization for Occhito sediment in the whole set of alkaline environments investigated here, cylindrical samples (diameter, 3 cm; height, 6 cm) were prepared by pouring the mixtures into polyethylene molds. Three samples were tested for each binding mixture. These samples were cured for 3 days at 60 °C in an oven, keeping the lids closed to ensure 100% relative humidity conditions. Afterwards, the specimens were extracted from the molds and kept in air for 11 days, until they were subjected to unconfined compressive strength (UCS) determination using a 100-kN capacity Controls MCC8 testing machine. Finally, in order to relate mechanical testing results with microstructural changes, SEM analysis was carried out by means of an FEI Quanta 200 FEG microscope.

## 4. Conclusions and Further Developments

In this paper, we reported a case study on the beneficial reuse of clay sediments coming from the dredging of Occhito Lake, located in Southern Italy. The problem of extending the life cycle of the dredged sediments by their sustainable reuse was approached here by considering geopolymer binder production. This management solution is favored by the push toward sustainability, which has involved also the construction industry and the promising results obtained in terms of mechanical performance, durability, fire resistance, *etc.* Furthermore, building material production in both precast and *in situ* applications represents a wide market, which could imply a positive trend, considering the huge amount of raw materials usually employed.

Sodium aluminate solution revealed to be the best activator, among those tested, in terms of mechanical property development (three days of compressive of about 7 MPa). In the case of this activator, calcination temperature can be beneficially reduced from 750 °C to 650 °C, implying further improvement of sustainability indicators. Other literature applications [23,24,25,26,27,28,29,30,31], as already pointed out, deal with a high temperature industrial process (about 1000–1150 °C). Calcination temperature reduction by means of binding system optimization represents a good way to improve sustainability indicators for the recycling process proposed here.

In a forthcoming experimental phase, starting from this initial, but wide research on binder kinetics and hardening properties, civil engineering composites (concrete, mortars and bricks) will be designed and characterized in terms of rheology, shrinkage and durability (freeze-thaw, sulfate attack, alkali silica reaction, chloride attack, *etc.*).
